# Urinary tubular biomarkers as predictors of death in critically ill patients with COVID-19

**DOI:** 10.2217/bmm-2021-0631

**Published:** 2022-05-09

**Authors:** Gabriela F Bezerra, Gdayllon C Meneses, Polianna LMM Albuquerque, Nicole C Lopes, Ranieri SS Santos, Juliana C da Silva, Sandra MB Mota, Rodrigo R Guimarães, Fábio R Guimarães, Álvaro R Guimarães, Caio MC Adamian, Paula R de Lima, Izabel CJ Bandeira, Márcia MP Dantas, Geraldo BS Junior, Reinaldo B Oriá, Elizabeth F Daher, Alice MC Martins

**Affiliations:** ^1^Post-Graduate Program in Pharmacology, School of Medicine, Universidade Federal do Ceará. Fortaleza, Ceará, 60430-275, Brazil; ^2^Medical Sciences Post-Graduate Program, Department of Internal Medicine, School of Medicine, Universidade Federal do Ceará. Fortaleza, Ceará, 60430-140, Brazil; ^3^Public Health Post-Graduate Program, School of Medicine, Health Sciences Center, Universidade de Fortaleza. Fortaleza, Ceará, 60811-905, Brazil; ^4^School of Medicine, Health Sciences Center, Universidade de Fortaleza. Fortaleza, Ceará, 60811-905, Brazil; ^5^Instituto José Frota (IJF) Hospital, Fortaleza, Ceará, 60025-061, Brazil; ^6^Laboratory of Tissue Healing, Ontogeny, & Nutrition, Department of Morphology, Institute of Biomedicine, Faculty of Medicine, Universidade Federal do Ceará, Fortaleza, Ceará, 60430-270, Brazil; ^7^Universidade Estadual do Ceará, Fortaleza, Ceará, 60714-903, Brazil; ^8^School of Medicine, Hospital Universitário Walter Cantídio, Universidade Federal do Ceará. Fortaleza, Ceará, 60430-372, Brazil

**Keywords:** COVID-19, death, kidney biomarkers, NGAL

## Abstract

**Aim:** To evaluate the prediction capacity of urinary biomarkers for death in critically ill patients with COVID-19. **Methods:** This is a prospective study with critically ill patients due to COVID-19 infection. The urinary biomarkers NGAL, KIM-1, MCP-1 and nephrin were quantified on ICU admission. **Results:** There was 40% of death. Urinary nephrin and MCP-1 had no association with death. Tubular biomarkers (proteinuria, NGAL and KIM-1) were predictors of death and cutoff values of them for death were useful in stratify patients with worse prognosis. In a multivariate cox regression analysis, only NGAL remains associated with a two-mount survival chance. **Conclusion:** Kidney tubular biomarkers, mostly urinary NGAL, had useful capacity to predict death in critically ill COVID-19 patients.

COVID-19 is an infectious disease caused by a novel coronavirus, which is named SARS-CoV-2 [1]. The initial symptoms include fever, cough, mild dyspnea, sore throat, headache, myalgia, blocked nose, diarrhea, vomiting and some cases evolved with severe complications, like an acute respiratory distress syndrome (ARDS), leading the patients to require placement in intensive care units (ICU) or even leading to death [2,3]. Among the risk factors associated with high mortality are advanced age, comorbidities such as diabetes, hypertension, obesity or immunosuppression and the need for invasive mechanical ventilation (IMV) [4–6]. More than 500 million cases of COVID-19 and 6 million deaths worldwide have been confirmed [7].

Kidney involvement in COVID-19 is common and has multifactorial causes, being associated with poor outcomes and death. Moreover, kidney disease is an independent risk factor for all-cause in-hospital death of COVID-19 patients [8]. Acute kidney injury (AKI) affects approximately 20–40% of patients with COVID-19 admitted to ICU, and it is a complication that has been linked to increased morbidity and mortality [9–11]. SARS-CoV-2 can invade and accumulate in the kidney causing directly endothelial damage. Moreover, the virus can infect the renal tubular epithelium and podocytes through an angiotensin converting enzyme 2 (ACE2)-dependent pathway [8,12–15]. Additionally, tubular damage may be associated to cytokine release syndrome (CRS), also known as ‘cytokine storm’ that occurs due to intrarenal inflammation, increased vascular permeability, volume depletion, and cardiomyopathy [16]. Other contributors to AKI include rhabdomyolysis, macrophage activation syndrome, and the development of microemboli and microthrombi in the context of hypercoagulability and endothelitis [13,17].

AKI complicates one in each five hospital admissions and was related with poor prognosis and increased health spending [18]. Currently, the gold standard in AKI diagnosis relies on increased serum creatinine and decreased urine production, which results in a rapid drop in the glomerular filtration rate. However, serum creatinine has low sensitivity and specificity and may not provide information of AKI etiology, prognosis, molecular pathways and treatment responses [18,19]. In addition, serum creatinine is a late marker of disease [20]. Indeed, more sensitive and specific biomarkers for AKI were warranted, because delays in detection and intervention have hindered clinical trials for COVID-19 treatment. Effective biomarkers could lead to early identification of AKI and predict progression for a higher disease severity.

Thus, assessing urinary biomarkers, which are easily quantifiable in urine, with better sensitivity and specificity, may be beneficial to the early detection of kidney-associated injury in COVID-19, and for preventive and therapeutic measures to halt AKI and ultimately death to patients. This study aims to evaluate the role of selected urinary biomarkers in predicting survival/death of patients with severe COVID-19.

## Methods

### Study design and selected patients

This is a prospective study of COVID-19 patients at the Instituto Doutor José Frota Hospital (IJF) in Fortaleza – Ceará, Brazil, from June 2020 to April 2021. The inclusion criteria were patients admitted to ICU of both genders, aged 18 years and older, who had a confirmed diagnosis by RT-PCR and who agreed to participate in the research and signed the free and informed consent form. Patients from medical wards and them who had ICU admission after wards were excluded. Patients with previous kidney disease and hospitalized admitted for reasons other than COVID-19, and the ones who acquired COVID during the hospital stay were also excluded.

After diagnosis, the blood and urine samples of the enrolled patients were collected on the time of hospital admission. These samples were centrifuged, aliquoted and frozen at -80°C until biomarkers analysis.

### Laboratory and clinical parameters of COVID-19 patients

Patients were followed-up during hospitalization through medical records to evaluate their kidney function and other clinical parameters. The severity of patients admitted to the ICU was estimated using the Simplified Acute Physiology Score 3 (SAPS3). This is a tool that uses data from patient admission to the ICU to assess the likelihood of death in the hospital outcome. Biochemical measurements of urinary creatinine (mg/dl) was done using an automatic biochemistry analyzer (Cobas C111, Roche^®^). Proteinuria was determined using the colorimetric method by reaction with pyrogallol red (Labtest^®^). To identify the presence or not of AKI, was used Kidney Disease Improving Global Outcome (KDIGO) criteria [21]. Briefly, was AKI stage 1 for patients who had serum creatinine (sCr) increases ≥0.3 mg/dl and up to twofold above the basal sCr. Increase in the range of two until threefold greater than the basal sCr were classified as AKI stage 2, and for AKI stage 3 the increase of the sCr more than threefold, as well as SCr values ≥4 mg/dl were considered.

### Urinary biomarkers measurements

The novel urinary kidney biomarkers were quantified using the ELISA, an immunoenzymatic assay. Commercial ELISA kits were acquired from the R&D Systems^®^ for NGAL (cat# DY1757), KIM-1 (cat# DY1750), MCP-1 (cat# DY279-05) and Nephrin (cat# DY4269). All urinary clinical markers and novel biomarkers evaluated were adjusted with urinary creatinine levels and expressed as a ratio of urinary creatinine to avoid bias of the urinary concentration [22].

### Statistical analysis

Categorical data were expressed as absolute counts and percentages. Chi-square test or Fisher exact test were used to evaluate the associations among categorical data, as appropriate according to expected frequencies values in 2 × 2 crosstabs. Quantitative data were first evaluated for normal distribution using Kolmogorov–Smirnov test. Normal data were expressed as mean ± standard deviation and non-normal data as median and interquartile range. Quantitative data were compared between two groups (discharge vs death patients) using Student's *t* test or a Mann–Whitney test as appropriate.

To evaluate predictive capacity for death of kidney biomarkers, ROC curves were constructed, and area under the ROC curve (AUC–ROC) with confidence intervals of 95% were calculated. The various cutoffs from ROC curve of each biomarker were investigated and determined using higher Youden index (Youden Index = sensitivity + specificity – 1). The selected cutoffs for predict death in COVID-19 patients were used to do new groups based on ‘lower than cutoff’ vs ‘higher than cutoff’.

These new groups were used to evaluate the survival behavior through Kaplan–Meier analysis for a 2-month survival chance. The log-rank test was used to evaluate the statistical difference between the two based on cutoff groups. Moreover, cox proportional hazards regression models were evaluated using urinary biomarkers cutoffs, previously associated parameters with death (using as condition p < 0.10) and other possible cofounds factors, such as comorbidities, were evaluated. Collinearity of variables was assessed. For multivariate model all selected variables were included manually, and was used a backward with likelihood ratio test as a stepwise method. Data were analyzed using SPSS software for Macintosh, version 23 (NY, USA; IBM Corp.). For all analytical tests, a base value of p < 0.05 was considered statistically significant.

### Ethics

This study was approved by the national committee on ethics in research, under CAAE number: 30579020.4.1001.0008. The patients were informed about the purpose of the study, and upon acceptance of participation, they signed the Free and Informed Consent form before the beginning of the evaluation.

## Results

### Characteristics of COVID-19 patients admitted in ICU

**Figure 1.**
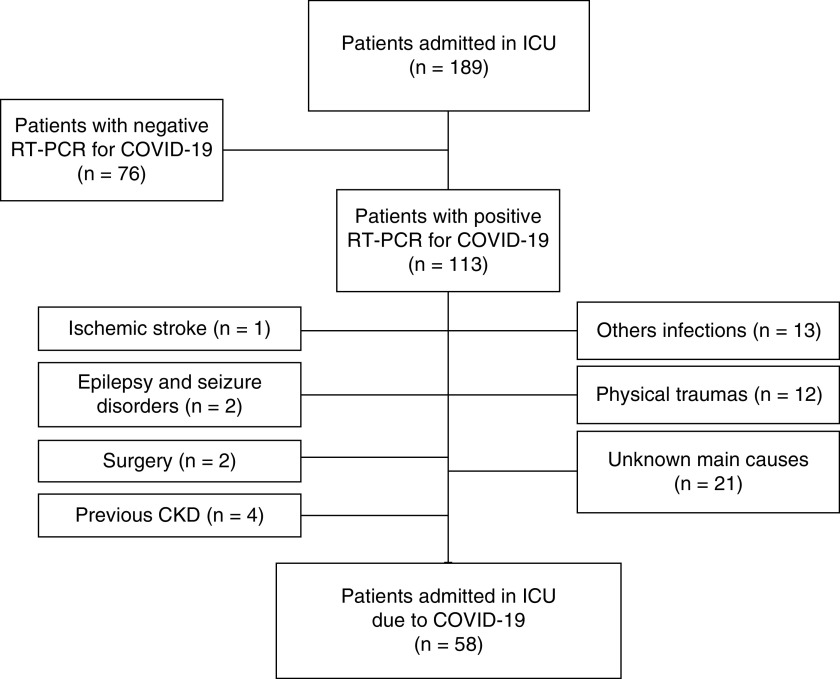
Flowchart of critically ill patients due to COVID-19 until final inclusion.

In total, 189 critically ill patients with COVID-19 had urinary samples collected. Among them, 76 patients had negative RT-PCR for COVID-19. Among 113 patients, 51 patients had other ICU admission main diagnosis rather than COVID-19 and were excluded. Moreover, four patients with previous CKD were excluded. Finally, 58 patients were selected with ICU admission due to COVID-19 infection as the leading cause (Figure 1).

In this group, 40 (69%) had AKI on the first week: 11 (19%) had KDIGO stage 1, 12 (21%) had stage 2 and 17 patients (29%) were stage 3. There was observed 38% of death. The male gender was predominant (59%), and the mean age was 57 ± 16 years. The comorbidities were present in 39 (67%) patients, including arterial hypertension (43%), obesity (38%), cardiopathy (32%), diabetes (29%) and asthma (6%). However, there was no significant statistical association between all of them with death. In the non-survivors group, the mean age was elevated compared to survivors (63 ± 13 vs 53 ± 16 years, p = 0.009) (Table 1).

**Table 1.** Epidemiologic characteristics of COVID-19 patients admitted on intensive care unit.

Parameter	Outcome			p-value^†^
	Total group (n = 58)	Survivors (n = 36)	Non-survivors (n = 22)	
Age (years)	56.6 ± 15.8	52.6 ± 16.3	63 ± 12.8	0.009
Gender				0.544
– Male	34 (58.6)	20 (55.6)	14 (63.6)	
– Female	24 (41.4)	16 (44.4)	8 (36.4)	
Time between symptoms until admission	10 (6–14)	10 (8–17)	8 (5–13)	0.079
Fever (adm)	38 (73.1)	23 (69.7)	15 (78.9)	0.469
Dyspnea	53 (91.4)	34 (94.4)	19 (86.4)	0.357
Oximetry	34 (59.6)	20 (57.1)	14 (63.6)	0.627
Comorbidity	39 (67.2)	22 (61.1)	17 (77.3)	0.203
– Cardiopathy	17 (32.1)	9 (26.5)	8 (42.1)	0.242
– Asthma	3 (5.7)	3 (8.8)	0 (0)	0.545
– Diabetes	16 (28.6)	9 (25)	7 (35)	0.427
– Neurologic	3 (5.7)	2 (5.9)	1 (5.3)	1.000
– Pneumopathy	2 (3.8)	1 (2.9)	1 (5.3)	1.000
– Obesity	21 (38.2)	12 (34.3)	9 (45)	0.431
– Hypertension	25 (43.1)	14 (38.9)	11 (50)	0.407

Quantitative data expressed as mean ± standard deviation or median and interquartile range between parenthesis according to distributions of the data. Qualitative data expressed as absolute count and percentages between parentheses.

† The chi-square test or Fisher exact test were applied for qualitative data and for quantitative data there was used Student t test or Mann–Whitney test according to normality.

### Comparison of clinical, laboratory and biomarkers according to death

Invasive respiratory support needed during ICU stay was more frequent in non-survivors than survivors patients (86% vs 56%, p = 0.019). Moreover, there was more frequency of vasopressors use (77% vs 42%, p = 0.008) and dialysis (59% vs 22%, p = 0.005) in non-survivors group. The SAPS3 score on ICU admission was elevated in the non-survivors patients (p = 0.001; Table 2).

**Table 2.** Comparison between clinical features, laboratory and biomarker results according to death in COVID-19 patients on admission and during ICU stay.

Parameter	Outcome		p-value^†^
	Survivors (n = 36)	Non-survivors (n = 22)	
**ICU admission parameters**			
Lowest mean arterial pressure	82 ± 14.5	73.6 ± 21	0.111
Highest heart rate	105.4 ± 19.9	110.5 ± 24.8	0.414
Highest respiratory rate	27.4 ± 7.2	29.6 ± 6.3	0.308
Highest arterial lactate	1.72 ± 0.79	1.6 ± 0.69	0.768
Lowest oxygenation index	142 (109–204)	135 (85–163)	0.499
SAPS3 score	51.4 ± 14.1	65.6 ± 14.6	0.001
**ICU stay parameters**			
Unit length stay (days)	13 (7.5–18.5)	8.5 (6–17)	0.118
Ventilatory support			0.019
– Invasive	18 (56.3)	19 (86.4)	
– Not invasive	14 (43.8)	3 (13.6)	
Dialysis	8 (22.2)	13 (59.1)	0.005
Vasopressors use	15 (41.7)	17 (77.3)	0.008
**Laboratory data on ICU admission**			
Hemoglobin (g/dl)	11.8 ± 2.2	11.7 ± 2.2	0.981
Leukocytes (per mm^3^)	12162 ± 4546	14478 ± 6479	0.130
Lymphocytes (per mm^3^)	806 (559–1087)	920 (602–1246)	0.824
Platelets (10^3^/mm^3^)	265 (208–313)	199 (150–302)	0.109
INR	1.08 ± 0.09	1.25 ± 0.47	0.052
aPTT	1.1 ± 0.25	1.35 ± 0.49	0.026
Serum urea (mg/dl)	39 (27–66)	57 (36–117)	0.049
Serum creatinine (mg/dl)	0.7 (0.6–1.05)	1.15 (0.9–1.5)	0.038
Serum potassium (mEq/l)	4 ± 1	4 ± 1	0.637
Serum sodium (mg/dl)	144 ± 6	145 ± 8	0.812
AST (U/l)	40 (27–67)	54 (34–72)	0.424
ALT (U/l)	49 (29–74)	39 (28–64)	0.35
LDH (U/l)	664 (547–881)	1027 (747–1299)	0.008
Total bilirubin (mg/dl)	0.54 (0.31–0.82)	0.58 (0.44–0.9)	0.917
C-reactive protein (pg/ml)	145.5 (35–194.4)	165.15 (127.1–248.2)	0.15
D-dimer (ng/ml)	2.27 (0.89–2.5)	2.1 (0.6–3.88)	0.813
**Urinary biomarkers on ICU admission**			
Proteinuria/creatinine ratio	0.6 (0.4–1.2)	1.5 (1–2.2)	0.004
Urinary NGAL (ng/mg-Cr)	91.9 (70.2–132.6)	148.3 (118.9–229.8)	0.002
Urinary MCP-1 (pg/mg-Cr)	2100.3 (1120.7–3436.5)	3284.9 (1315.7–5072.2)	0.088
Urinary nephrin (pg/mg-Cr)	1227 (679.7–1910.3)	1802.9 (862.8–3294.4)	0.102
Urinary KIM-1 (pg/mg-Cr)	1440.5 (826.4–2362.9)	3278.8 (1861.9–5238.6)	0.006

Quantitative data expressed as mean ± standard deviation or median and interquartile range between parenthesis according to distributions of the data. Qualitative data expressed as absolute count and percentages between parentheses.

† The chi-square test or Fisher's exact test were applied for qualitative data and for quantitative data there was used Student's *t* test or Mann–Whitney test according to normality.

Regarding ICU admission laboratory parameters, there was no statistical difference between hemoglobin, leukocytes, lymphocytes, potassium, sodium, aspartate aminotransferase (AST), alanine aminotransferase (ALT), c-reactive protein (CRP), total bilirubin and D-dimer according to death. However, the non-survivors group presented in ICU admission impairment of coagulation factors, such as higher activated partial thromboplastin time (aPTT) compared to survivors patients. Conventional kidney biomarkers were elevated in non-survivors group on ICU admission through serum creatinine (1.15 [0.9–1.5] vs 0.7 [0.6–1.05], p = 0.038) and serum urea (57 [36–117] vs 39 [27–66], p = 0.049). Also, lactic dehydrogenase (LDH) levels were higher in non-survivors group (1027 [747–1299] vs 664 [547–881], p = 0.008) (Table 2).

**Figure 2.**
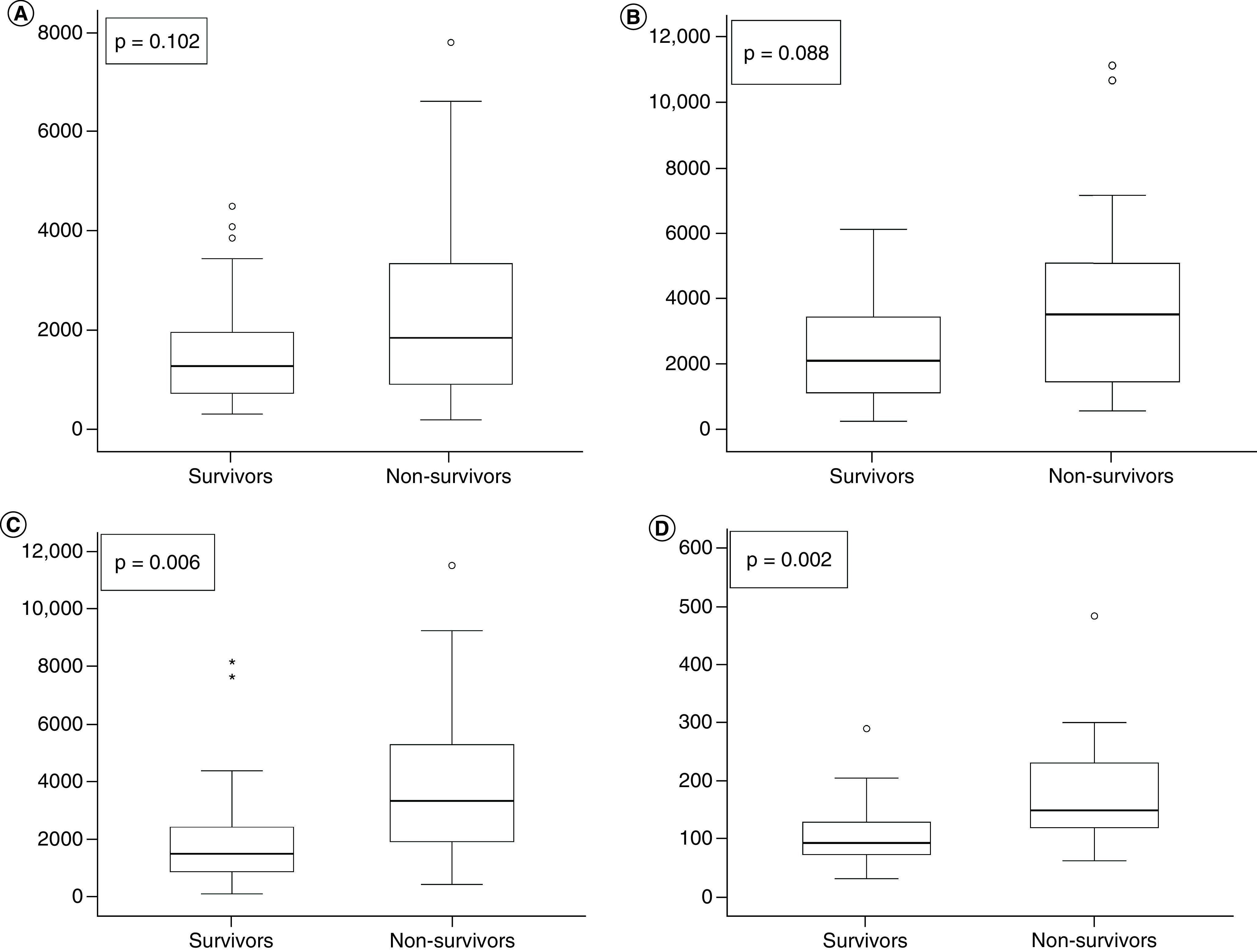
Box-plot of each urinary biomarkers levels evaluated on ICU admission of COVID-19, according to death during hospital stay. **(A)** Urinary nephrin; **(B)** urinary MCP-1; **(C)** urinary KIM-1; **(D)** urinary NGAL.

All urinary biomarkers of the non-survivors group had high levels on ICU admission. However, biomarkers related to glomerular structural changes and inflammation, urinary nephrin and MCP-1, had no statistical significance with death. On the other hand, in non-survivors group, elevated levels on ICU admission of proteinuria/creatinine ratio (1.5 [1–2.2] vs 0.6 [0.4–1.2], p = 0.004) and specific renal tubular biomarkers, such as urinary NGAL (148.3 [118.9–229.8] vs 91.9 [70.2–132.6] ng/mg-Cr, p = 0.002) and urinary KIM-1 (3278 [1861–5238] vs 1440 [826–2362] pg/mg-Cr, p = 0.006) had statistical significance (Figure 2).

### Predictive values of urinary biomarkers for death

**Figure 3.**
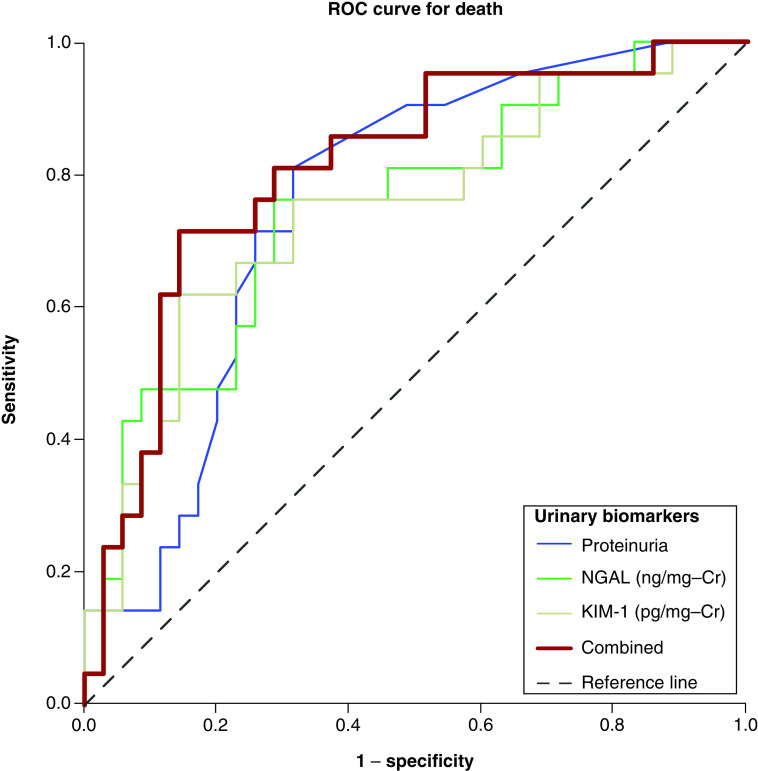
ROC curve analysis of urinary biomarkers for death of COVID-19 patients.

The conventional (serum creatinine and urea) and urinary biomarkers with previous statistical significance (p < 0.05) for death in comparisons (proteinuria, KIM-1 and NGAL) were selected for ROC curve analysis to predict death. There was observed that proteinuria, urinary KIM-1 and urinary NGAL had better AUC–ROC (0.728; p = 0.004, 0.749; p = 0.002 and 0.750; p = 0.002, respectively) (Table 3). In a combined approach using proteinuria*KIM-1*NGAL, the AUC–ROC improves for 0.810; p < 0.001 (Table 3 & Figure 3).

**Table 3.** Predictive values of selected urinary biomarkers quantified on ICU admission for death of COVID-19 patients.

Parameter	Cutoff	Sensitivity(%)	Specificity(%)	AUC–ROC (CI 95%)	p-value
Serum urea (mg/dl)	52	68	67	0.659 (0.512–0.807)	0.043
Serum creatinine (mg/dl)	0.85	77	67	0.713 (0.570–0.856)	0.007
Proteinuria/creatinine ratio	0.90	77	67	0.728 (0.593–0.862)	0.004
Urinary NGAL (ng/mg-Cr)	118	76	71	0.750 (0.616–0.883)	0.002
Urinary KIM-1 (ng/mg-Cr)	1.81	77	70	0.749 (0.616–0.881)	0.002
Combined (NGAL, KIM-1 and proteinuria) (ng/mg-Cr)	379	71	86	0.810 (0.691–0.928)	<0.001

AUC–ROC: Area under the ROC curve.

Urinary NGAL cutoff of the 118 ng/mg-Cr had 76% of sensitivity and 71% of specificity. Urinary KIM-1 cutoff of the 1.81 ng/mg-Cr had 77% of sensitivity and 70% of specificity. Proteinuria/creatinine ratio cutoff of 0.90 had 77% of sensitivity and 67% of specificity. In combined proteinuria*KIM-1*NGAL, the cutoff of the 379 (ng/mg-Cr) had 71% of sensitivity and 86% of specificity (Table 3).

### Survival analysis and Cox proportional-hazards regression models using urinary biomarkers for 2-month survival chance

**Figure 4.**
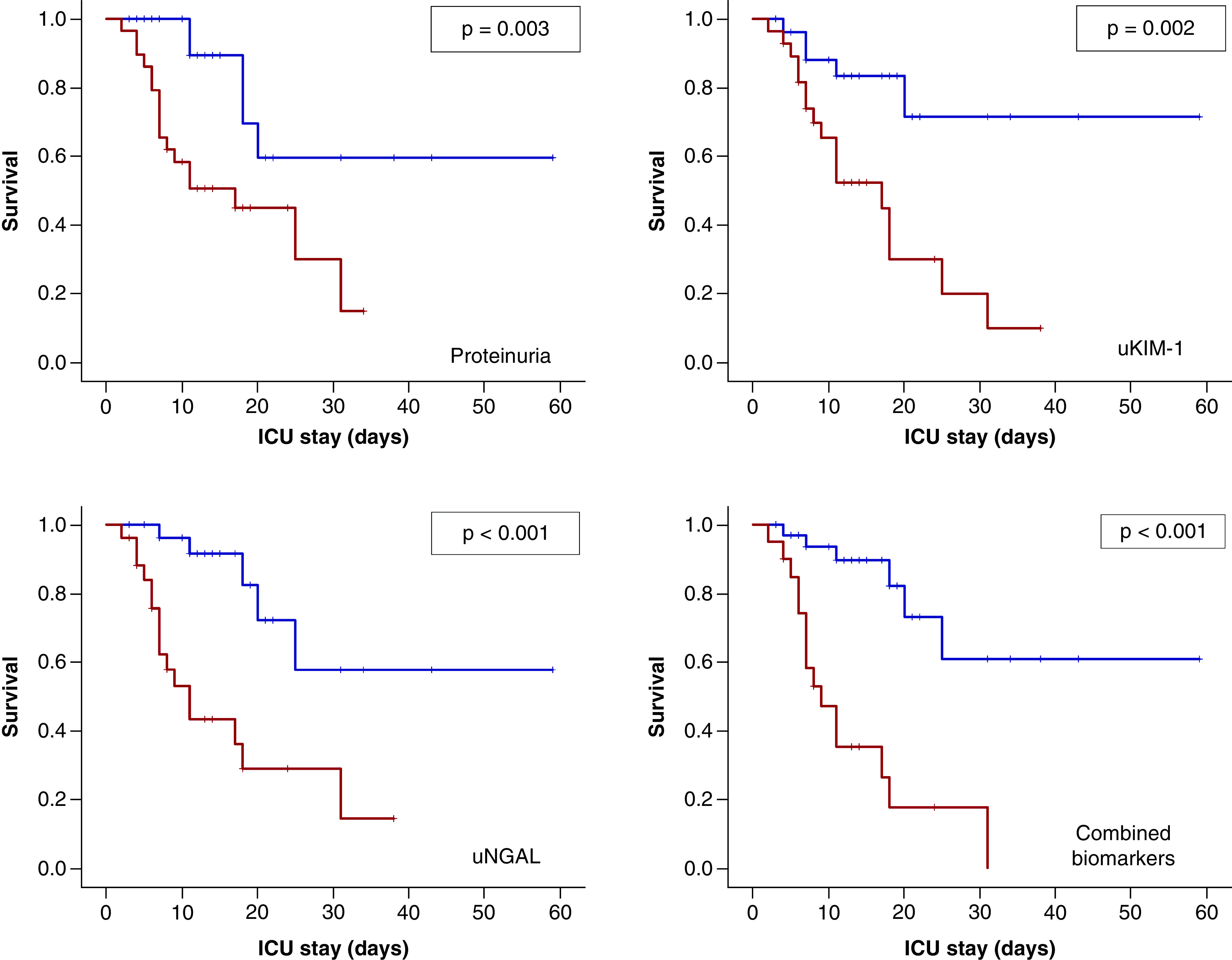
Analysis for 2-month survival with comparisons using previous determined cutoff values of each urinary biomarker. Red curve: COVID-19 patients with urinary levels higher than cutoff. Blue curve: COVID-19 patients with urinary levels lower than cutoff.

The COVID-19 patients were stratified according to ‘lower than’ vs ‘higher than’ previously selected cutoff of each urinary biomarker. Patients with values higher than cutoff in all urinary biomarkers died more quickly with statistical significance using log-rank test (Figure 4).

In Cox regression analysis, ICU admission parameters associated with two-mount survival chance in the univariate analysis include age, SAPS3 score, proteinuria/creatinine ratio (>0.90), urinary KIM-1 (>1.8 ng/mg-Cr), and urinary NGAL (>118.8 ng/mg-Cr). In multivariate models adjusted for age, gender, SAPS3, LDH (U/l), dialysis, comorbidities, proteinuria's cutoff, urinary KIM-1's cutoff, urinary NGAL's cutoff and vasopressors use, among urinary biomarkers, only urinary NGAL remains statistically significant with two-mount survival chance with hazard ratio = 5.666 (CI 95%: 1.761–18.227), p = 0.004, in the final model (Table 4).

**Table 4.** Cox proportional-hazards regression models using urinary biomarkers for 2-month survival chance.

Parameter	Death		p-value
	Hazard ratio	CI 95%	
**Unadjusted**			
Age (per each 10 years)	1.521	1.076–2.150	0.018
SAPS3 (per each 30 points)	2.422	1.131–5.183	0.023
Dialysis	2.021	0.859–4.756	0.107
Vasopressors use	2.033	0.743–5.563	0.167
Proteinuria/creatinine ratio (>0.90)	3.962	1.441–10.698	0.007
Urinary KIM-1 (>1.8 ng/mg-Cr)	4.252	1.563–11.565	0.005
Urinary NGAL (>118.8 ng/mg-Cr)	5.363	1.954–14.718	0.001
**Multivariate model** ^†^			
Gender (male)	3.341	0.887–12.579	0.075
Dialysis	3.380	1.156–9.885	0.026
Urinary NGAL (>118.8 ng/mg-Cr)	5.666	1.761–18.227	0.004

† Adjusted for age, gender, SAPS3, LDH, Dialysis, comorbidities, proteinuria's cutoff, urinary KIM-1's cutoff, urinary NGAL's cutoff, and vasopressors use.

AUC–ROC: Area under the ROC curve.

## Discussion

This present study addressed the level of urinary biomarkers related to glomerular or tubular changes in severe COVID-19 patients and its early association with death. This cohort study documented a strong association between kidney tubular damage and mortality in COVID-19 in the intensive care setting. Assessing urinary biomarkers on hospital admission in these patients might provide additional value in predicting not just kidney impairment but the risk of death.

There are many pathways that could lead to kidney damage following SARS CoV-2 infection, such as inflammatory and immune systemic responses, endothelial injury, activation of the coagulation cascade, disrupted renin–angiotensin system and pathological crosstalk between lung and kidney [23]. In this present study, the greatest need for ventilatory support (mainly mechanical ventilation), dialysis and vasopressors were related with multiorgan failure in severe COVID-19, as have been reported in others studies [11,24]. Acute hypoxemia might interfere in kidney function and increase renal vascular resistance, which might contribute to renal hypoperfusion and acute tubular injury [25,26]. As with all instances of multiorgan failure, there is a high mortality rate and need for mechanical ventilatory support in severe patients with COVID-19 [23]. Complex crosstalk between lung and kidney has been reported in severe patients with COVID-19. Indeed, the mechanical ventilatory support with high positive end-expiratory pressure (PEEP) increases the risk of AKI in severe patients, leading to higher mortality, too [27]. Moreover, the renal blood flow may be more compromised in SARS-Cov-2 patients than in acute respiratory distress syndrome following other illnesses [25].

This present study observed kidney proximal and possible distal tubular damage due to elevated levels of proteinuria, urinary NGAL and KIM-1 in patients with COVID-19 who had worse prognosis and died more quickly. Proteinuria also had important predictive values for death in COVID-19 patients, mainly when evaluated with kidney tubular damage biomarkers (NGAL and KIM-1) in the combined analysis.

The proteinuria is common in COVID-19 and has been reported even in the absence of AKI and may denote a subclinical insult [28,29]. Proteinuria results from glomerular membrane barrier permeability dysfunction or impaired reabsorption by epithelial cells of proximal tubuli [30]. Most reports strongly support the notion that acute tubular injury is the primary lesion driving the kidney injury following the COVID-19 [31]. According to autopsy studies, acute tubular injury is by far the most common finding in kidneys of patients with COVID-19 [23]. Some studies have reported mild proteinuria, which corroborates with tubular dysfunction. However, tubular proteinuria has been associated with mortality [28]. Unfortunately, proteinuria is well recognized as a marker of chronic kidney disease (CKD) and its progression, being a risk factor for cardiovascular events and death among both the general and CKD populations [30]. In fact, in this present study, isolated and combined proteinuria with tubular biomarkers were more reliable to predict the death of the patients with COVID-19 with no previous CKD.

The KIM-1, a molecule upregulated upon kidney injury, is a potential receptor for SARS-CoV-2 [32]. KIM-1 is expressed in lung and kidney epithelial cells in COVID-19 patients and is a receptor for SARS-CoV-2 [32]. Furthermore, higher urine concentrations of KIM-1 were independently associated with death and could be associated with proximal tubule injury and implicated in ischemia-reperfusion injury in kidney [33]. Among multiorgan manifestations in COVID-19 patients, apart from the lung, the kidney is highly vulnerable, and renal dysfunctions are strongly associated with mortality [32]. A previous study has related tubular markers, such as KIM-1 and N-acetyl-beta-glucosaminidase, with AKI and severe disease in COVID-19 patients. Moreover, the overexpression of the KIM-1 during SARS-CoV 2 invasion could provide insights into potential therapeutic targets. Assessing KIM-1 on the admission of patients with COVID-19 might provide additional value in predicting mortality and improve the early management of the clinical complications.

This current study confirmed that a high urinary NGAL level is a potential strong predictor of a high risk of in-hospital death, even after adjusting for parameters well recognized for death in COVID-19 and other possible cofounds factors. Urinary NGAL is a novel biomarker of renal tubular injury and has presented good sensibility and accuracy in detecting AKI in several settings, and elevated levels of urinary NGAL were more associated with proximal and distal tubular injury [34]. Studies with systemic and urinary NGAL as predictors of AKI evidenced the importance of avoiding mortality before surgery and other interventions [35]. A recent study validated a prognostic model of AKI and in-hospital death in the same setting using NGAL and artificial intelligence, and presented a similar conclusion [36]. Hence, this present study suggests important targets of kidney dysfunction along tubular tissue during COVID-19 infection and its impact on survival in the context of severe COVID-19 profile. This present discovery supports the use of NGAL as a valuable biomarker that may improve earlier diagnosis and better assess risk groups.

On the other hand, in this present study, biomarkers related to glomerular dysfunction such as urinary nephrin and MCP-1 had no association with mortality. Urinary MCP-1 and nephrin may be a proxy of glomerular impairment, which plays an important role in kidney diseases and has been studied in diverse scenarios [37,38]. The glomerular damage in COVID-19 is not common and has been described in patients with *APOL1* genotypes, related to collapsing glomerulopathy in black patients [39]. This present study corroborates with this hypothesis, being the evident kidney tubular damage detected and associated with death rather than glomerular injury.

## Limitations

This is a single-center study with small sample size, even though several effects could be detected. Some clinical data, such as the previous comorbidities and medications, such as nephrotoxic agents, were lacking. The sample size was compromised with almost half of critically ill patients excluded, because other concomitant causes could interfere with the death outcome rather than only COVID-19 infection complications. The 24-h collection that would help to correct factors such as hypovolemia was not performed. The changes in biomarkers after admission was not evaluated, and as the indication for hospitalization of patients with COVID-19 depends on the facilities, data at the time of admission alone may not be sufficient.

## Conclusion

Kidney tubular biomarkers were markedly higher in the non-survival patients, being the urinary NGAL an independent predictor of the death in COVID-19. Also, severe COVID-19 infection affects mostly kidney tubular cells rather than glomerular structure.

**Summary points**

**Background**

- Kidney involvement in COVID-19 is common and is an independent risk factor for all-cause in-hospital death of COVID-19 patients.
- Assessment of novel urinary biomarkers may be promising way for the early detection of COVID-19 complications, improving patients care and ultimately decreasing mortality.

**Methods**

- A prospective study with critically ill COVID-19 patients, and their urine was collected in the first hours of ICU admission for kidney biomarkers quantification.
- The prediction performance of urinary biomarkers for death during ICU stay was evaluated using ROC curve and Kaplan–Meier analysis with Cox regression.

**Results**

- Elevated levels of kidney tubular biomarkers, such as proteinuria, urinary NGAL and urinary KIM-1, were associated with poor prognosis and mortality of COVID-19 patients in the intensive care setting.
- Urinary NGAL was an independent predictor for the death of COVID-19 in multivariate Cox regression.

**Conclusions**

- Severe COVID-19 infection may primarily affect kidney tubular cells rather than glomerular structure.
- Assessing urinary biomarkers, mostly urinary NGAL, on hospital admission might provide additional value in predicting kidney impairment as well as the risk of death.

## References

[1] Yang P, Wang X. COVID-19: a new challenge for human beings. Springer Nature 17, 555–557 (2020). 10.1038/s41423-020-0407-x PMC7110263 32235915

[2] Pinto B, Pinto Moehlecke Iser-Av José Acácio Moreira B. Suspected COVID-19 case definition: a narrative review of the most frequent signs and symptoms among confirmed cases. Epidemiol. Serv. Saude, Brasília 29(3), 2020 (2020). 10.5123/S1679-49742020000300018 32609142

[3] Hu B, Huang S, Yin L. The cytokine storm and COVID-19. John Wiley and Sons Inc. 93, 250–256 (2021). 10.1002/jmv.26232 PMC7361342 32592501

[4] Grasselli G, Greco M, Zanella A Risk factors associated with mortality among patients with COVID-19 in intensive care units in Lombardy, Italy Supplemental content. JAMA Intern. Med. 180(10), 1345–1355 (2020). 32667669 10.1001/jamainternmed.2020.3539 PMC7364371

[5] Albitar O, Ballouze R, Ooi JP, Maisharah S, Ghadzi S. Risk factors for mortality among COVID-19 patients. Diabetes Res. Clin. Pract. 166, 108293 (2020). 32623035 10.1016/j.diabres.2020.108293 PMC7332436

[6] Zhou F, Yu T, Du R Clinical course and risk factors for mortality of adult inpatients with COVID-19 in Wuhan, China: a retrospective cohort study. Lancet 395(10229), 1054–1062 (2020). 32171076 10.1016/S0140-6736(20)30566-3 PMC7270627

[7] WHO Coronavirus (COVID-19) Dashboard | WHO Coronavirus Disease (COVID-19) Dashboard. CEST, 29 April 2022. https://covid19.who.int

[8] Ronco C, Reis T, Husain-Syed F. Management of acute kidney injury in patients with COVID-19. Lancet Respir. Med. 8(7), 738–742 (2020). 32416769 10.1016/S2213-2600(20)30229-0 PMC7255232

[9] SARS-CoV-2 renal tropism associates with acute kidney injury. Lancet 396(10251), 597–598 (2020). 32818439 10.1016/S0140-6736(20)31759-1 PMC7431179

[10] Acute kidney injury in COVID-19 patients | LIVES 2020.

[11] Richardson S, Hirsch JS, Narasimhan M Presenting characteristics, comorbidities, and outcomes among 5700 patients hospitalized with COVID-19 in the New York City Area. J. Am. Med. Assoc. 323(20), 2052–2059 (2020). 10.1001/jama.2020.6775 PMC7177629 32320003

[12] Su H, Yang M, Wan C Renal histopathological analysis of 26 postmortem findings of patients with COVID-19 in China. Kidney Int. 98(1), 219–227 (2020). 32327202 10.1016/j.kint.2020.04.003 PMC7194105

[13] Varga Z, Flammer AJ, Steiger P Endothelial cell infection and endotheliitis in COVID-19. Lancet Publishing Group 395, 1417–1418 (2020). 10.1016/S0140-6736(20)30937-5 PMC7172722 32325026

[14] Diao B, Wang C, Wang R Human kidney is a target for novel severe acute respiratory syndrome coronavirus 2 (SARS-CoV-2) infection. medRxiv 12(1), 2506 (2020). 10.1038/s41467-021-22781-1 PMC8096808 33947851

[15] Cheng Y, Luo R, Wang K Kidney disease is associated with in-hospital death of patients with COVID-19. Kidney Int. 97(5), 829–838 (2020). 32247631 10.1016/j.kint.2020.03.005 PMC7110296

[16] Ronco C, Reis T. Kidney involvement in COVID-19 and rationale for extracorporeal therapies. Nat. Rev. Nephrol. 16(6), 308–310 (2020). 32273593 10.1038/s41581-020-0284-7 PMC7144544

[17] Zhang Y, Xiao M, Zhang S Coagulopathy and antiphospholipid antibodies in patients with Covid-19. N. Engl. J. Med. 382(17), e38 (2020). 32268022 10.1056/NEJMc2007575 PMC7161262

[18] Moledina DG, Parikh CR. Phenotyping of acute kidney injury: beyond serum creatinine. Semin Nephrol. 38(1), 3–11 (2018). 29291759 10.1016/j.semnephrol.2017.09.002 PMC5753429

[19] Waikar SS, Betensky RA, Emerson SC, Bonventre JV. Imperfect gold standards for kidney injury biomarker evaluation. J. Am. Soc. Nephrol. 23(1), 13–21 (2012). 22021710 10.1681/ASN.2010111124 PMC3695762

[20] Lima C, Macedo E. Urinary biochemistry in the diagnosis of acute kidney injury. Dis. Markers 2018, 4907024 (2018). 30008975 10.1155/2018/4907024 PMC6020498

[21] Kellum JA, Lameire N, Aki K, Work G. Art: 10.1186/Cc11454. (Part 1), 17, 1–15 (2013).

[22] Waikar SS, Sabbisetti VS, Bonventre JV. Normalization of urinary biomarkers to creatinine during changes in glomerular filtration rate. Kidney Int. 78(5), 486–494 (2010). 20555318 10.1038/ki.2010.165 PMC3025699

[23] Legrand M, Bell S, Forni L Pathophysiology of COVID-19-associated acute kidney injury. Nat. Rev. Nephrol. 17(11), 751–764 (2021). 34226718 10.1038/s41581-021-00452-0 PMC8256398

[24] Grasselli G, Zangrillo A, Zanella A Baseline characteristics and outcomes of 1591 patients infected with SARS-CoV-2 admitted to ICUs of the Lombardy Region, Italy. JAMA 323(16), 1574 (2020). 32250385 10.1001/jama.2020.5394 PMC7136855

[25] Fogagnolo A, Grasso S, Dres M Focus on renal blood flow in mechanically ventilated patients with SARS-CoV-2: a prospective pilot study. J. Clin. Monit. Comput. 36(1), 161–167 (2021). 33385260 10.1007/s10877-020-00633-5 PMC7775615

[26] Darmon M, Schortgen F, Vargas F Diagnostic accuracy of Doppler renal resistive index for reversibility of acute kidney injury in critically ill patients. Intensive Care Med. 37(1), 68–76 (2011). 20862450 10.1007/s00134-010-2050-y

[27] Ottolina D, Zazzeron L, Trevisi L Acute kidney injury (AKI) in patients with Covid-19 infection is associated with ventilatory management with elevated positive end-expiratory pressure (PEEP). J. Nephrol. 35(1), 99–111 (2021). 34170508 10.1007/s40620-021-01100-3 PMC8226340

[28] Huart J, Bouquegneau A, Lutteri L Proteinuria in COVID-19: prevalence, characterization and prognostic role. J. Nephrol. 34(2), 355–364 (2021). 33484426 10.1007/s40620-020-00931-w PMC7823174

[29] Mohamed MMB, Velez JCQ. Proteinuria in COVID-19. Clin. Kidney J. 14(Suppl. 1), i40–i47 (2021). 33815781 10.1093/ckj/sfab036 PMC7995522

[30] Luis J, Martinez-castelao A. Proteinuria: detection and role in native renal disease progression. Transplant. Rev. 26(1), 3–13 (2012). 10.1016/j.trre.2011.10.002 22137726

[31] Mohamed MMB, Lukitsch I, Torres-Ortiz AE Acute kidney injury associated with coronavirus disease 2019 in Urban New Orleans. Kidney 360 1(7), 614–622 (2020). 35372932 10.34067/KID.0002652020 PMC8815549

[32] Yang C, Zhang Y, Zeng X Kidney injury molecule-1 is a potential receptor for SARS-CoV-2. J. Mol. Cell Biol. 13(3), 185–196 (2021). 33493263 10.1093/jmcb/mjab003 PMC7928767

[33] Vanmassenhove J, Vanholder R, Nagler E, Van Biesen W. Urinary and serum biomarkers for the diagnosis of acute kidney injury: an in-depth review of the literature. Nephrol. Dial. Transplant. 28(2), 254–273 (2013). 23115326 10.1093/ndt/gfs380

[34] Kuwabara T, Mori K, Mukoyama M Urinary neutrophil gelatinase-associated lipocalin levels reflect damage to glomeruli, proximal tubules, and distal nephrons. Kidney Int. 75(3), 285–294 (2009). 19148153 10.1038/ki.2008.499

[35] de Geus HRH, Ronco C, Haase M, Jacob L, Lewington A, Vincent J-L. The cardiac surgery-associated neutrophil gelatinase-associated lipocalin (CSA-NGAL) score: a potential tool to monitor acute tubular damage. J. Thorac. Cardiovasc. Surg. 151(6), 1476–1481 (2016). 26952930 10.1016/j.jtcvs.2016.01.037

[36] He L, Zhang Q, Li Z Incorporation of urinary neutrophil gelatinase-associated lipocalin and computed tomography quantification to predict acute kidney injury and in-hospital death in COVID-19 patients. Kidney Dis. 7(2), 120–130 (2020). 10.1159/000511403 PMC7573910 33824868

[37] Munshi R, Johnson A, Siew ED MCP-1 gene activation marks acute kidney injury. J. Am. Soc. Nephrol. 22(1), 165–75 (2011). 21071523 10.1681/ASN.2010060641 PMC3014045

[38] Kostovska I, Trajkovska T, Topuzovska S Urinary nephrin is earlier, more sensitive and specific marker of diabetic nephropathy than microalbuminuria. J. Med. Biochem. 39(1), 83–90 (2019). 10.2478/jomb-2019-0026 PMC7282240 32549781

[39] Wu H, Larsen CP, Hernandez-Arroyo CF AKI and Collapsing glomerulopathy associated with COVID-19 and APOL 1 high-risk genotype. J. Am. Soc. Nephrol. 31(8), 1688–1695 (2020). 32561682 10.1681/ASN.2020050558 PMC7460910

